# Synthesis of New 1*H*-1,2,3-Triazole Analogs in Aqueous Medium *via* “*Click*” Chemistry: A Novel Class of Potential Carbonic Anhydrase-II Inhibitors

**DOI:** 10.3389/fchem.2021.642614

**Published:** 2021-06-30

**Authors:** Satya Kumar Avula, Majid Khan, Sobia Ahsan Halim, Ajmal Khan, Samia Ahmed Al-Riyami, Rene Csuk, Biswanath Das, Ahmed Al-Harrasi

**Affiliations:** ^1^ Natural and Medical Sciences Research Center, University of Nizwa, Nizwa, Oman; ^2^ H.E.J. Research Institute of Chemistry, International Center for Chemical and Biological Sciences, University of Karachi, Karachi, Pakistan; ^3^ Organic Chemistry, Martin-Luther-University Halle-Wittenberg, Halle, Germany

**Keywords:** synthesis, 1H-1, 2, 3-triazole analogs, click chemistry, aqueous medium, carbonic anhydrase-II inhibitory activity, molecular docking studies

## Abstract

A series of novel 1*H*-1,2,3-triazole analogs (**9a–j**) were synthesized *via “Click”* chemistry and Suzuki–Miyaura cross-coupling reaction in aqueous medium. The compounds were evaluated for their carbonic anhydrase-II enzyme inhibitory activity *in vitro*. The synthesis of triazole **7a** was accomplished using (*S*)-(-) ethyl lactate as a starting material. This compound (**7a)** underwent Suzuki–Miyaura cross-coupling reaction with different arylboronic acids in aqueous medium to afford the target molecules, **9a–j** in good yields. All newly synthesized compounds were characterized by ^1^H NMR, ^13^C NMR, FT-IR, HRMS, and where applicable ^19^F NMR spectroscopy (**9b**, **9e**, **9h**, and **9j**). The new compounds have shown moderate inhibition potential against carbonic anhydrase-II enzyme. A preliminary structure-activity relationship suggested that the presence of polar group at the 1*H*-1,2,3-triazole substituted phenyl ring in these derivatives (**9a–j**) has contributed to the overall activity of these compounds. Furthermore, *via* molecular docking, it was deduced that the compounds exhibit inhibitory potential through direct binding with the active site residues of carbonic anhydrase-II enzyme. This study has unraveled a new series of triazole derivatives as good inhibitors against carbonic anhydrase-II.

## Introduction

1*H*-1,2,3-Triazole molecules play a vital role in pharmaceuticals and agrochemicals (Abdel-Wahab et al., 2012). The triazole moiety is very important in organic chemistry due to its broad range of applications in biomedicinal, biochemical, and material sciences (Singh et al., 2010). The chemistry of the compounds containing this moiety underwent substantial growth over the past decades (Thirumurugan et al., 2013). These compounds are widely used in industrial applications such as dyes, photographic materials, photostabilizers, agrochemicals, and corrosion inhibitors (copper alloys) (Fan et al., 1996).

**FIGURE 1:**
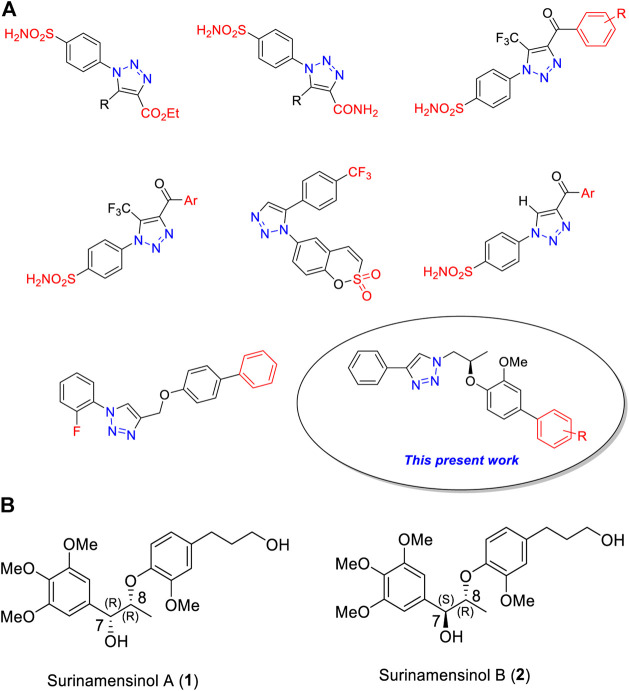
**(A)** Chemical structures of some clinically used 1*H*-1,2,3-triazole analogs as potential carbonic anhydrase-II inhibitors. **(B)** Chemical structures of surinamensinols A and B.

Recent literature studies (Kumar et al., 2018; Sharma et al., 2019) demonstrated that 1*H*-1,2,3-triazole ring containing heterocycles was showing superior carbonic anhydrase inhibitors (Figure 1A).

Carbonic anhydrase (CAs, EC 4.2.1.1), a Zn^+2^ containing metallic enzyme, catalyzes the reversible reaction of carbon dioxide into bicarbonate ions (Supuran and De Simone, 2015; Ozensoy and Guler et al., 2016). There are fifteen isoforms of CA which have been identified so far (Lindskog, 1997; Aggarwal et al., 2013). They possess a difference in their organ distributions, levels of gene expression, molecular sequence features, and kinetic parameters (Krishnamurthy et al., 2008). CAs are key contributors to various physiological and pathological processes. Thus, they are considered the prime therapeutic target for the treatment of several chronic diseases.

In continuation of our research work on 1*H*-1,2,3-triazole derivatives (Avula et al., 2018; Avula et al., 2019), we herein report a new series of 1*H*-1,2,3-triazole analogs (**9a–j**) as carbonic anhydrase-II inhibitors (Huisgen, 1963; Pham Thi et al., 2016). We have selected the 8th triazole structure (Figure 1A) for our present synthesis because it contains a chiral dioxyaryl moiety which is present in some bioactive natural products such as surinamensinols A and B (Figure 1B) (add two references). A structural-activity relationship is discussed to demonstrate the influence of structural moieties on the triazole derivatives.

## Results and Discussion

### Chemistry: Synthesis of 1*H*-1,2,3-Triazole Analogs (9a-j)

The first step employed Mitsunobu reaction between (*S*)-(-) ethyl lactate **1** and 4-bromo-2-methoxy phenol using diisopropylazodicarboxylate (DIAD). The reaction afforded the expected compound **2** in 86% yield. In this step, a chiral center has been successfully introduced. Reduction of compound **2** to compound **3** was achieved by using DIBAL-H which furnished compound **3** in 90% yield. The hydroxyl group of **3** was converted into a tosyl moiety to provide compounds **4** in high yield (95%). The latter was treated with NaN_3_ and afforded the corresponding azide derivative **5** in 78% yield.

**SCHEME 1:**
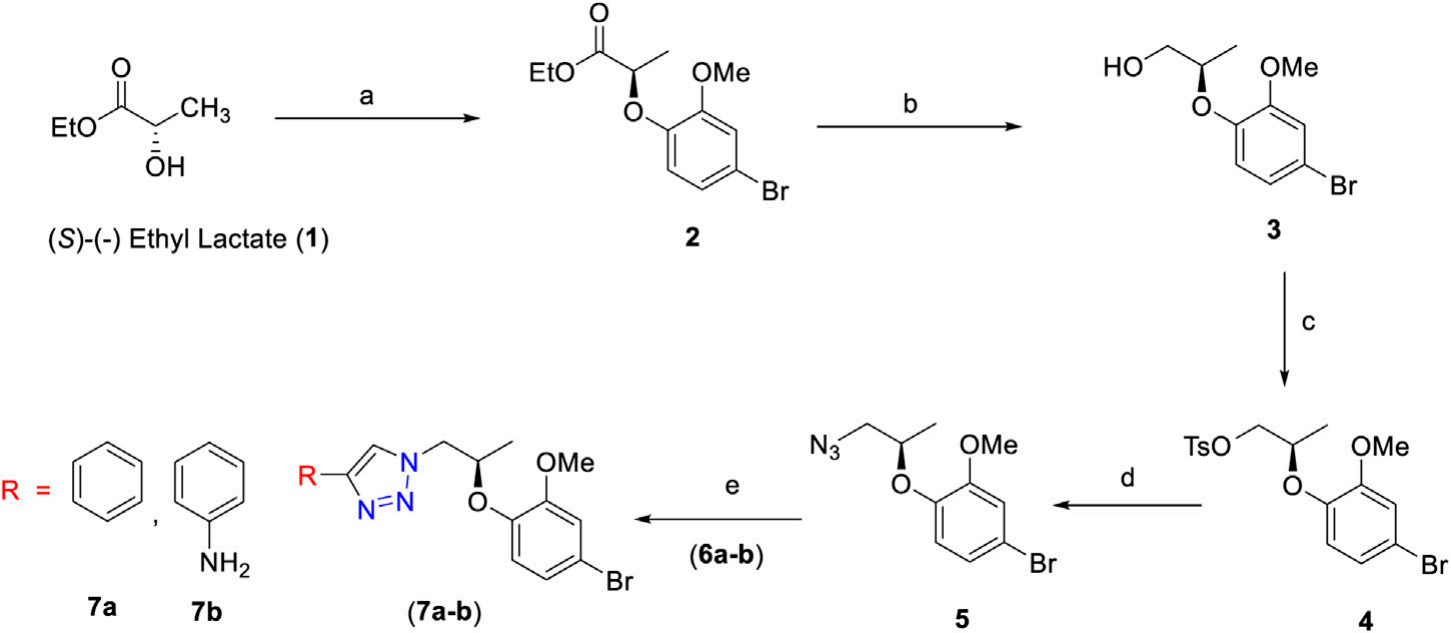
Reagents and conditions: **(A)** 4-bromo-2-methoxyphenol, PPh_3_, DIAD, dry THF, 0°C to room temperature, 3 h, 86%; **(B)** DIBAL-H, dry DCM, 0°C to room temperature, 3 h, 90%; **(C)** TsCl, Et_3_N, dry DCM, DMAP, 0°C to room temperature, 5 h, 95%; **(D)** NaN_3_, DMF, 70°C, 3 h, 78%; **(E)** alkyne derivative (**6a–b**), CuI, Et_3_N, MeCN, room temperature, 3 h, **7a** (76%) and **7b** (82%).

Azide **5** underwent 1,3-dipolar cycloaddition with the alkyne derivative **6a** in the presence of CuI and Hunig’s base. The reaction furnished the desired product 1*H*-1,2,3-triazole derivative **7a** as a colorless amorphous solid in 76% yield. The ^1^H NMR spectrum of compound **7a** showed singlet at δ 8.05 for triazole proton (–CH–N_3_). The eight aromatic protons appeared in the region of δ 7.78–6.63 ppm. One multiplet and doublet of doublet signals at δ 4.61–4.56 and δ 4.48 correspond to the–CH–O– and –CH–N–, respectively. A singlet peak at δ 3.69 is due to methoxy protons on the phenyl ring and a doublet at δ 1.24 is attributed to methyl protons of –CH–O–Ph. The high-resolution mass spectrometric data at 388.0661 (M^+^) support the structure of compound **7a**. Similarly, using the same reaction conditions described for the synthesis of compound **7a**, compound **7b** was obtained in 82% yield using different alkyne derivative (**6b**). The synthesis of compounds **7a** and **7b** is summarized in Scheme 1.

**SCHEME 2:**
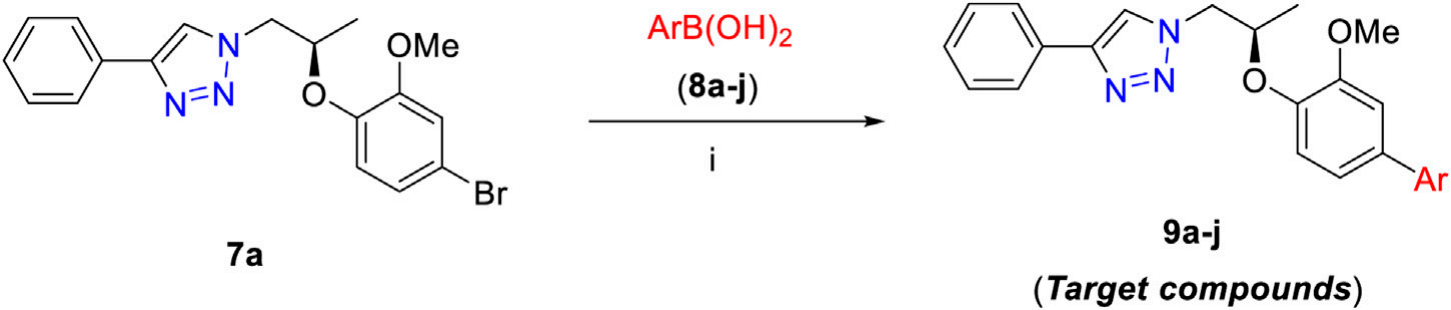
Synthesis of **9a–j**. Reagents and conditions: (i) **7a** (1.0 equiv), **8a–j** (1.2–1.5 equiv), Pd(OAc)_2_ (5 mol%), K_2_CO_3_ (3.0 equiv), THF:H_2_O (3 : 1), 80–85°C 10–12 h (82–91%).

The final step of this series was Suzuki–Miyaura cross-coupling reaction (Shaulis et al., 2002; Polshettiwar et al., 2010) of compound **7a** with different arylboronic acids (**8a–j)** to afford a novel series of 1*H*-1,2,3-triazole analogs (**9a–j**) in good yields (82–91%) (Scheme 2, Table 1). Here, both electron-withdrawing and electron-donating arylboronic acids were successfully employed. The best yields were obtained in THF : H_2_O (3 : 1) at 85–90°C for 10–12 h using Pd(OAc)_2_ as a catalyst (5 mol%) and K_2_CO_3_ (3.0 equiv) as a base. The structures of all the new synthesized compounds (**9a–j**) were confirmed by spectroscopic techniques (^1^H NMR, ^13^C NMR, FT-IR, HRMS, and where applicable ^19^F NMR). The synthesis of a novel series of 1*H*-1,2,3-triazole analogs (**9a–j**) is summarized in Scheme 2.

**TABLE 1:** Synthesis of cross-coupled 1*H*-1,2,3-triazole analogs (**9a–j**).

Reagents (8)	Compounds (9)	Ar	Yield of 9 (%)^a^
A	**a**	Ph	82
B	**b**	3.5-(F_3_C)_2_C_6_H_3_	90
C	**c**	4-AcC_6_H_4_	82
D	**d**	3-NO_2_C_6_H_4_	85
E	**e**	2-(F_3_C)C_6_H_4_	83
F	**f**	4-MeC_6_H_4_	82
G	**g**	4-MeOC_6_H_4_	84
H	**h**	4-FC_6_H_4_	89
I	**i**	4-ClC_6_H_4_	87
J	**j**	2.6-(F)_2_C_6_H_3_	91

^a^ Yields refer to pure isolated products.

### Anticarbonic Anhydrase-II Activities of 1*H*-1,2,3-Triazole Analogs (9a–j)

**FIGURE 2:**
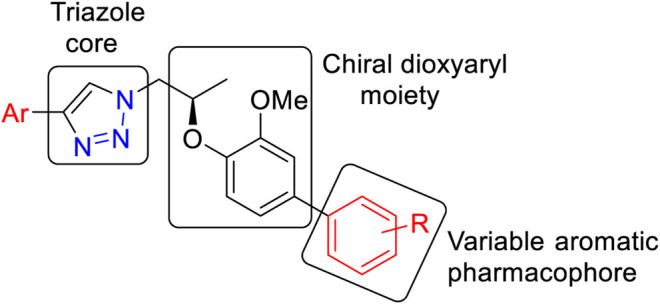
General structural feature of the synthesized molecules (**9**).

The general structural features of the synthesized molecules (**9**) are presented in Figure 2. Two pharmacophoric elements (triazole and chiral dioxyaryl) were considered as a rigid motif with an aryl group attached to the chiral dioxyaryl moiety which is mainly determining the varying degree of activity. A diverse array of functional groups (both electropositive and electronegative) influencing the activity toward carbonic anhydrase-II inhibition has been utilized.

All synthesized 1*H*-1,2,3-triazole analogs (**9a–j)** were evaluated against bovine carbonic anhydrase-II enzyme to know their therapeutic potential. All compounds showed potent to significant activities with IC_50_ values in the range of 13.8–35.7 µM, as compared to standard acetazolamide (18.2 ± 0.23 µM). In *in vitro* carbonic anhydrase-II assay, compounds **7b** (13.8 ± 0.63 µM) and **9e** (18.1 ± 1.31 µM) showed potent activities, followed by **9d** (20.7 ± 1.13 µM), **9c** (21.5 ± 0.21 µM), **9b** (25.1 ± 1.04 µM), and **9f** (26.6 ± 0.80 µM), while compounds **9h** (32.3 ± 0.77 µM) and **9j** (35.7 ± 0.57 µM) demonstrated lower activities. The detailed predictive SAR was deduced from molecular docking studies. The *in vitro* and docking results are tabularized in Table 2.

**TABLE 2:** *In vitro* and *in silico* results of the compounds against carbonic anhydrase-II.

Compounds	IC_50_ ± SEM (µM)	Score	Binding interaction			
			Ligand atoms	Receptor atoms	Interaction type	Distance (Å)
7b	13.8 ± 0.63	−6.93	N23	ZN	Metallic	2.92
			N23	OG1-THR199	HBD	2.54
			N23	N-THR199	HBD	1.64
			O15	ND2-ASN62	HBD	2.15
			N11	ND2-ASN62	HBD	3.00
				HOH270	HBD	3.37
9e	18.1 ± 1.31	−6.30	N2	NE2-GLN92	HBD	2.53
			O3	ND2-ASN62	HBD	3.03
9d	20.7 ± 1.13	−5.37	O31	ZN	Metallic	2.78
			O32	N-THR199	HBA	2.53
			5-ring	NE2-HIS64	π-cation	2.62
9c	21.5 ± 0.51	−5.24	O29	ZN	Metallic	2.50
9b	25.1 ± 1.04	−5.21	N15	NE2-GLN92	HBA	2.50
9f	26.6 ± 0.80	−4.98	6-ring	NE2-HIS64	π-cation	2.73
			6-ring	N-THR199	π-π	2.58
9h	32.3 ± 0.77	−4.52	6-ring	NE1-TRP5	π-H	3.70
			N16	ND2-ASN62	HBA	2.82
9j	35.7 ± 0.57	−4.05	6-ring	NE2-HIS64	π-cation	2.82
7a	NA	—	—	—	—	—
9a	NA	—	—	—	—	—
9g	NA	—	—	—	—	—
9i	NA	—	—	—	—	—
Acetazolamide	18.2 ± 0.23	−5.40	O10	ZN	Metallic	2.77
	ZN		O11	OG1-THR198	HBA	2.90

HBA, hydrogen bond acceptor; HBD, hydrogen bond donor.

### Molecular Docking Studies and Predicted Structure-Activity Relationship

**FIGURE 3:**
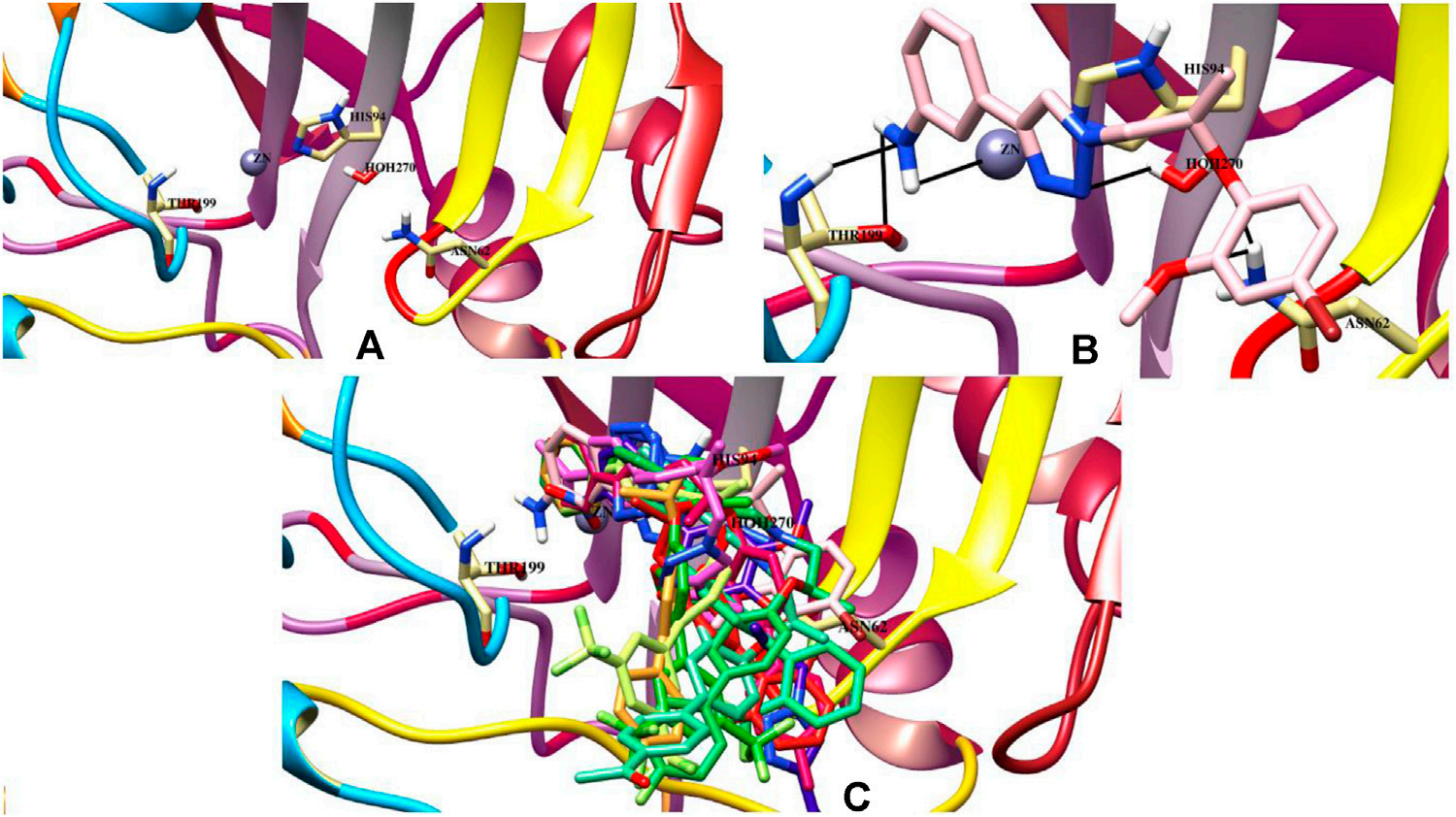
**(A)** The active site of the carbonic anhydrase is shown. **(B)** The binding mode of the most active compound (**7b**) within the active site of carbonic anhydrase. Ligand is presented as a light pink stick model and hydrogen bonds are shown in black lines. **(C)** The docked poses of all active ligands in the active site of the bovine carbonic anhydrase.

**FIGURE 4:**
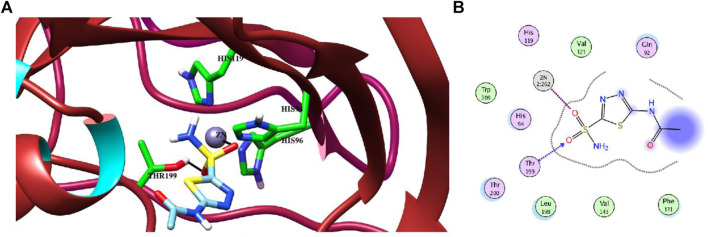
**(A)** The binding mode of the standard inhibitor “acetazolamide (AZM)” in the active site of CA-II in the 3D form. H-bond and metal-ligand interaction are shown in black lines. **(B)** The protein-ligand interaction is shown in 2D form. The H-bond and metal-ligand interaction are displayed in blue and magenta dotted lines, respectively.

Molecular docking studies of all active triazole derivatives were performed using molecular operating software (MOE) (Chemical Computing Group, 2014), in order to determine the best plausible binding modes of the ligands in the active site of the enzyme. The active site of CA-II is depicted in Figure 3A. Compound **7b** (IC_50_ = 13.8 ± 0.63 µM) was found to be the most active compound of the series and its best predicted binding pose is presented in Figure 3B. The binding interaction of **7b** demonstrated that its aniline moiety formed a metallic bond with Zn^+2^ ion and hydrogen bonding with the amino and –OH groups of Thr199. Furthermore, the dioxyaryl group of **7b** exhibited H-bonding with the amide group of Asn62. Additionally, a water molecule (HOH270) also offered an H-bond to the triazole moiety of **7b**. This multiple bonding of the compound with the active site residues is responsible for the enhanced biological activity of **7b** as compared to the rest of the compounds. Similarly, the triazole nitrogen and methoxy oxygen of the **9e** (IC_50_ = 18.1 ± 1.31 µM) interacted with the side chains of Gln92 and Asn62, respectively. The loss of hydrogen bond donor/acceptor group at triazole substituted aryl group of **9e** makes the molecule less active than **7b**; moreover, water molecule does not contribute to protein-ligand bridging for **9e**. The nitro group of **9d** mediated H-bonding and metallic interaction with the amide group of Thr199 and Zn ion, respectively. Additionally, the side chain of His64 provided π-cation interaction to the triazole ring of **9d**. The dioxyaryl group does not interact with the surrounding residues including Asn62, Asn67, Gln92, and water molecules; this may be the reason for lower activity of **9d** than **7b** and **9e**. The docked view of **9c** showed that the carbonyl oxygen interacted with the Zn ion *via* metallic bond; however, the other polar groups do not interact with the active site residues and the docked orientation of **9c** is surface exposed. This is the reason for the further reduced activity of **9c**. Similarly, the triazole substituted aryl group of **9b** does not possess any polar moiety to interact with Zn ion or Thr199 and His94. However, the triazole nitrogen of **9b** formed H-bond with the side chain of Gln92. The dioxyaryl moiety and its substituted R group of **9b** remained surface exposed, which further decreased the inhibitory activity of **9b**. Likewise, the triazole substituted aryl group and dioxyaryl substituted aryl group of **9f** mediated hydrophobic interactions with the side chains of Thr199 and His64, respectively, while the triazole ring and the dioxyaryl group lost interaction with Gln92 and Asn62, respectively. Due to the loss of these interactions, the compound exhibited less biological activity than **9b**. The binding mode of **9h** was similar to the docked view of **9f**; however, the triazole nitrogen and the dioxyaryl substituted aryl group of **9h** formed H-bonding and hydrophobic interaction with Asn62 and Trp5, respectively. Similarly, the least active compound, **9j**, exhibited only π-cation interaction with the side chain of His64, while its triazole ring and the dioxyaryl group do not interact with the surrounding residues; due to the loss of major H-bonding or metallic interactions, the compound exhibited the least inhibitory activity against CA-II. The best-docked poses of the active compounds are depicted in Figure 3C. The docking scores and the binding interactions are tabulated in Table 2. Acetazolamide was used as a positive control in docking which exhibited biological activity with IC_50_ value of 18.2 µM. The sulfate group of acetazolamide interacted with the Zn ion and the side chain of Thr198 through metallic interaction and H-bonding, respectively. The docking score of acetazolamide is −5.40, which is lesser than the docking scores of **7b** and **9e**, while being greater than the docking scores of **9d**, **9c**, **9b**, **9f**, **9h**, and **9j**. The docked view of acetazolamide is shown in Figure 4 in both 3D and 2D format. The docking scores and binding interactions of the compounds are well correlated with the inhibitory activities of the compounds. The docked orientation of compounds showed that Thr199 and Zn ions play important role in the stabilization of the triazole substituted aryl group, while the dioxyaryl group interacts with Asn62 or Gln92. Additionally, His64 provides hydrophobic interactions to the compounds.

## Conclusion

In summary, a series of novel 1*H*-1,2,3-triazole analogs were synthesized (**9a–j**) and evaluated for their carbonic anhydrase-II inhibitory activity *in vitro*. (*S*)-(-) Ethyl lactate was used as a starting material to introduce the chirality of the target molecules. The triazole moiety was prepared via “*Click*” chemistry and the aryl derivatives by utilizing Suzuki–Miyaura cross-coupling reaction in aqueous medium. All the compounds have shown moderate inhibition potential against carbonic anhydrase-II enzyme reported for the first time. The molecular docking studies showed that all the active compounds well accommodate to the active site of the CA enzyme.

## Experimental Section

### General

Reagents were obtained from Sigma-Aldrich, Germany. Silica gel for column chromatography was of 100–200 mesh. Solvents were purified by following standard procedures. Thin-layer chromatography (TLC) was carried using silica gel F_254_ precoated plates. UV-light and I_2_ stain were used to visualize the spots. The ^1^H and ^13^C NMR spectra were recorded on NMR spectrometer (Bruker: 600 MHz for ^1^H, 150 MHz for ^13^C, and 564 MHz for ^19^F) using CDCl_3_ as a solvent. The high-resolution electrospray ionization mass spectra (HR-ESI-MS) were recorded on Agilent 6530 LC Q-TOF instrument. Organic extracts and solutions of pure compounds were dried over anhydrous MgSO_4_.

### General Procedure for Preparation of (R)-1-[(1-Azidopropan-2-yl)oxy]-4-bromo-2-methoxybenzene (Azide Compound 5)

The key intermediate azide compound **5** was prepared according to our earlier literature method (Avula et al., 2018).

### General Procedure for Synthesis of 1*H*-1,2,3-Triazole Derivatives (7a and 7b)

CuI (2.0 equiv) and triethylamine (3.0 equiv) were added to a solution of azide compound **5** (1.0 equiv) and alkyne derivatives **6a–b** (1.2 equiv) in acetonitrile (10 ml) at room temperature, and the mixture was stirred for 3 h. The reaction mixture was diluted with EtOAc (20 ml), 10 ml of aqueous NH_4_Cl was added, the aqueous layer was extracted with EtOAc (3 × 15 ml), and the combined organic layer was washed with brine solution, dried over anhydrous Na_2_SO_4_, and concentrated in vacuo to obtain a crude residue that was purified by flash chromatography to obtain desired 1*H*-1,2,3-triazole derivatives (**7a**) 76% and (**7b**) 82%.

#### (R)-1-(2-(4-Bromo-2-methoxyphenoxy)propyl)-4-phenyl-1H-1,2,3-triazole (**7a**)

Colorless amorphous solid; Yield = 76%; IR (solid): 1498, 1265, 1128, 732 cm^−1^; ^1^H NMR (600 MHz, chloroform-*d*) δ 8.05 (s, 1H), 7.78 (d, *J* = 7.7 Hz, 2H), 7.35 (t, J = 7.6 Hz, 2H), 7.25 (d, J = 7.6 Hz, 1H), 6.92–6.87 (m, 2H), 6.63 (d, J = 8.4 Hz, 1H), 4.61–4.56 (m, 2H), 4.48 (dd, J = 14.8, 7.5 Hz, 1H), 3.69 (s, 3H), 1.24 (d, J = 6.2 Hz, 3H); ^13^C NMR (150 MHz, chloroform-*d*) δ 151.5, 147.4, 130.8, 128.8, 128.0, 125.5, 123.5, 121.5, 119.2, 115.6, 115.0, 75.1, 55.9, 54.8, 17.1; HRMS (ESI^+^): Found (M+H^+^): 388.0661 C_18_H_19_^79^BrN_3_O_2_ required 388.0664. Found (M+H^+^): 390.0642 C_18_H_19_^81^BrN_3_O_2_ required 390.0639.

#### (R)-4-(1-(2-(4-Bromo-2-methoxyphenoxy)propyl)-1H-1,2,3-triazol-4-yl)aniline (**7b**)

Brown color solid; Yield = 82%; IR (solid): 3380, 1586, 1496, 1268, 1130, 842, 736 cm^−1^; ^1^H NMR (600 MHz, DMSO-*d*_6_) δ 7.90 (s, 1H), 7.43 (d, *J* = 8.0 Hz, 2H), 6.88–6.83 (m, 2H), 6.65 (d, *J* = 8.5 Hz, 1H), 6.59 (d, *J* = 8.1 Hz, 2H), 4.55 (ddd, *J* = 29.5, 12.1, 4.9 Hz, 2H), 4.46 (dd, *J* = 14.2, 6.8 Hz, 1H), 4.23 (brs, 2H), 3.67 (s, 3H), 1.20 (d, *J* = 6.5 Hz, 3H); ^13^C NMR (150 MHz, DMSO-*d*_6_) δ 151.5, 145.5, 126.5, 123.4, 120.1, 119.1, 115.7, 114.9, 114.7, 75.0, 56.0, 54.6, 17.2; HRMS (ESI^+^): Found (M+H^+^): 404.2002 C_24_H_24_^79^BrN_3_O_2_ required 404.2004. Found (M+H^+^): 406.0714 C_24_H_24_^81^BrN_3_O_2_ required 406.0716.

### General Suzuki–Miyaura Reaction Procedure for Synthesis of Cross-Coupled 1H-1,2,3-Triazole Analogs in Aqueous Medium (**9a–j**)

Different substituted boronic acids **8a–j** (1.2–1.5 equiv per crossing coupling step), K_2_CO_3_ (3.0 equiv), and Pd(OAc)_2_ (5 mol%) were added to a mixture of phenyl 1H-1,2,3-triazole 7a (1.0 equiv), THF : H_2_O (3 : 1, 5 ml). The reaction was heated at 80–85°C with stirring under a nitrogen atmosphere for 10–12 h, cooled to room temperature, and diluted with water (5 ml). The resulting reaction mixture was extracted with Et_2_O. The organic layers were combined and washed with brine solution, dried over anhydrous MgSO_4_, and then filtered off. The solvent was removed under vacuum, and the crude product was purified by silica gel column chromatography (eluting with EtOAc/hexane, 9 : 1) to give pure cross-coupled products **9a–j**.

#### (R)-1-(2-((3-Methoxy-[1,1′-biphenyl]-4-yl)oxy)propyl)-4-phenyl-1H-1,2,3-triazole (**9a**)

White solid; Yield = 82%; IR (solid): 1498, 1214, 774, 746, 667 cm^−1^; ^1^H NMR (600 MHz, chloroform-*d*) δ 8.16 (s, 1H), 7.83–7.79 (m, 2H), 7.52–7.46 (m, 2H), 7.39 (td, *J* = 7.7, 2.8 Hz, 4H), 7.29 (t, *J* = 7.4 Hz, 2H), 7.10–7.01 (m, 2H), 6.88 (d, *J* = 8.1 Hz, 1H), 4.77–4.69 (m, 2H), 4.59 (dd, *J* = 14.4, 7.2 Hz, 1H), 3.85 (s, 3H), 1.37 (d, *J* = 6.2 Hz, 3H); ^13^C NMR (150 MHz, chloroform-*d*) δ 150.6, 147.4, 145.4, 140.5, 136.3, 130.6, 128.6, 127.8, 126.9, 126.7, 125.4, 121.4, 119.4, 117.7, 111.1, 75.0, 55.6, 54.8, 17.1; HRMS (ESI^+^): Found (M+H^+^): 386.1856 C_24_H_24_N_3_O_2_ required 386.1854.

#### (R)-1-(2-((3-Methoxy-3′,5′-bis(trifluoromethyl)-[1,1′-biphenyl]-4-yl)oxy)propyl)-4-phenyl-1H-1,2,3-triazole (**9b**)

Colorless amorphous solid; Yield = 90%; IR (solid): 1496, 1328, 1267, 1168, 1219, 774 cm^−1^; ^1^H NMR (600 MHz, chloroform-*d*) δ 8.14 (s, 1H), 7.90 (s, 2H), 7.81 (d, *J* = 8.3 Hz, 3H), 7.41 (t, *J* = 7.6 Hz, 2H), 7.34–7.28 (m, 1H), 7.09–7.02 (m, 2H), 6.93 (d, *J* = 8.2 Hz, 1H), 4.86–4.72 (m, 2H), 4.62 (dd, *J* = 14.3, 7.3 Hz, 1H), 3.90 (s, 3H), 1.41 (d, *J* = 6.2 Hz, 3H); ^13^C NMR (150 MHz, chloroform-*d*) δ 151.1, 147.5, 147.0, 142.8, 133.1, 132.1, 131.9, 130.6, 128.7, 128.0, 126.8, 125.5, 124.2, 122.4, 121.5, 120.5, 119.9, 117.6, 111.1, 75.0, 56.0, 55.0, 17.2; ^19^F NMR (564 MHz, chloroform-*d*): δ −62.82; HRMS (ESI^+^): Found (M+H^+^): 522.1627 C_26_H_22_F_6_N_3_O_2_ required 522.1630.

#### (R)-1-(3′-Methoxy-4′-((1-(4-phenyl-1H-1,2,3-triazol-1-yl)propan-2-yl)oxy)-[1,1′-biphenyl]-4-yl)ethan-1-one (**9c**)

Pale yellow amorphous solid; Yield = 82%; IR (solid): 1680, 1498, 1217, 842, 754, 671 cm^−1^; ^1^H NMR (600 MHz, chloroform-*d*) δ 8.11 (s, 1H), 7.87 (s, 2H), 7.83 (d, *J* = 8.3 Hz, 2H), 7.79 (d, *J* = 8.6 Hz, 3H), 7.39 (t, *J* = 7.7 Hz, 2H), 7.05 (d, *J* = 8.6 Hz, 1H), 6.85 (d, *J* = 8.3 Hz, 2H), 4.81–4.70 (m, 2H), 4.60 (dd, *J* = 14.3, 7.2 Hz, 1H), 3.88 (s, 3H), 2.50 (s, 3H), 1.39 (d, *J* = 6.3 Hz, 3H); ^13^C NMR (150 MHz, chloroform-*d*) δ 195.3, 152.3, 145.9, 144.2, 137.4, 132.5, 130.7, 128.8, 128.0, 127.5, 126.9, 125.6, 125.1, 121.4, 120.6, 119.9, 115.6, 115.3, 111.1, 75.1, 56.0, 55.0, 17.3; HRMS (ESI^+^): Found (M +H^+^): 428.1973 C_26_H_26_N_3_O_3_ required 428.1971.

#### (R)-1-(2-((3-Methoxy-3′-nitro-[1,1′-biphenyl]-4-yl)oxy)propyl)-4-phenyl-1H-1,2,3-triazole (**9d**)

Light brown amorphous solid; Yield = 85%; IR (solid): 1598, 1496, 1343, 1216, 842, 771, 747, 669 cm^−1^; ^1^H NMR (600 MHz, chloroform-*d*) δ 8.09 (s, 1H), 7.63 (dd, *J* = 12.4, 7.7 Hz, 2H), 7.54 (d, *J* = 7.8 Hz, 1H), 7.44 (t, *J* = 8.0 Hz, 2H), 7.39 (t, *J* = 7.6 Hz, 3H), 7.29 (s, 1H), 6.93 (d, *J* = 8.4 Hz, 2H), 6.67 (d, *J* = 8.2 Hz, 1H), 4.65 (td, *J* = 13.7, 4.7 Hz, 2H), 4.54 (dd, *J* = 13.8, 6.7 Hz, 1H), 3.74 (s, 3H), 1.30 (d, *J* = 6.1 Hz, 3H); ^13^C NMR (150 MHz, chloroform-*d*) δ 151.5, 147.5, 145.3, 132.8, 132.1, 132.0, 130.6, 129.6, 128.8, 128.6, 128.5, 128.1, 125.6, 123.6, 121.6, 119.2, 115.6, 115.2, 75.3, 55.9, 54.9, 17.1; HRMS (ESI^+^): Found (M+H^+^): 431.1732 C_24_H_23_N_4_O_4_ required 431.1735.

#### (R)-1-(2-((3-Methoxy-2′-(trifluoromethyl)-[1,1′-biphenyl]-4-yl)oxy)propyl)-4-phenyl-1H-1,2,3-triazole (**9e**)

White amorphous solid; Yield = 83%; IR (solid): 1498, 1329, 1263, 1164, 1219, 845, 772, 667 cm^−1^; ^1^H NMR (600 MHz, chloroform-*d*) δ 8.08 (s, 1H), 7.79 (d, *J* = 7.8 Hz, 2H), 7.60 (dd, *J* = 12.7, 7.5 Hz, 2H), 7.45 (d, *J* = 8.0 Hz, 2H), 7.40 (d, *J* = 7.7 Hz, 2H), 7.31 (d, *J* = 7.5 Hz, 1H), 6.95 (d, *J* = 7.3 Hz, 2H), 6.68 (d, *J* = 8.5 Hz, 1H), 4.69–4.62 (m, 2H), 4.54 (dd, *J* = 13.9, 6.9 Hz, 1H), 3.74 (s, 3H), 1.30 (d, *J* = 6.1 Hz, 3H); ^13^C NMR (150 MHz, chloroform-*d*) δ 151.5, 147.6, 145.3, 133.7, 132.1, 132.0, 130.9, 130.5, 129.2, 128.8, 128.6, 128.5, 128.1, 125.6, 123.6, 121.6, 119.2, 115.6, 115.2, 75.3, 55.9, 54.9, 17.1; ^19^F NMR (564 MHz, chloroform-*d*): δ −59.28; HRMS (ESI^+^): Found (M+H^+^): 454.1744 C_25_H_23_F_3_N_3_O_2_ required 454.1742.

#### (R)-1-(2-((3-Methoxy-4′-methyl-[1,1′-biphenyl]-4-yl)oxy)propyl)-4-phenyl-1H-1,2,3-triazole (**9f**)

White solid; Yield = 82%; IR (solid): 1496, 1217, 845, 753, 669 cm^−1^; ^1^H NMR (600 MHz, chloroform-*d*) δ 8.16 (s, 1H), 7.81 (d, *J* = 7.6 Hz, 2H), 7.39 (t, *J* = 7.4 Hz, 4H), 7.29 (t, *J* = 7.4 Hz, 1H), 7.18 (s, 2H), 7.05–7.01 (m, 2H), 6.86 (d, *J* = 8.1 Hz, 1H), 4.74–4.68 (m, 2H), 4.58 (dd, *J* = 14.7, 7.4 Hz, 1H), 3.84 (s, 3H), 2.35 (s, 3H), 1.36 (d, *J* = 6.1 Hz, 3H); ^13^C NMR (150 MHz, chloroform-*d*) δ 150.8, 147.5, 145.4, 137.8, 136.9, 136.5, 130.8, 129.4, 128.7, 127.9, 126.7, 125.6, 121.6, 119.3, 118.0, 111.1, 75.1, 55.7, 55.0, 21.0, 17.3; HRMS (ESI^+^): Found (M+H^+^): 400.2017 C_25_H_26_N_3_O_2_ required 400.2020.

#### (R)-1-(2-((3,4′-Dimethoxy-[1,1′-biphenyl]-4-yl)oxy)propyl)-4-phenyl-1H-1,2,3-triazole (**9g**)

Pale yellow amorphous solid; Yield = 84%; IR (solid): 1497, 1239, 1214, 770, 744, 668 cm^−1^; ^1^H NMR (600 MHz, chloroform-*d*) δ 8.18 (d, *J* = 1.1 Hz, 1H), 7.84–7.80 (m, 2H), 7.45–7.39 (m, 4H), 7.33–7.29 (m, 1H), 7.04–7.00 (m, 2H), 6.96–6.92 (m, 2H), 6.88 (d, *J* = 8.1 Hz, 1H), 4.76–4.71 (m, 2H), 4.60 (dd, *J* = 14.7, 7.4 Hz, 1H), 3.85 (d, *J* = 1.1 Hz, 3H), 3.83 (d, *J* = 1.1 Hz, 3H), 1.38 (d, *J* = 6.1 Hz, 3H); ^13^C NMR (150 MHz, chloroform-*d*) δ 158.9, 150.8, 147.5, 145.1, 136.2, 133.3, 130.8, 128.7, 127.9, 125.6, 121.5, 119.0, 118.0, 114.1, 110.9, 75.1, 55.7, 55.3, 54.9, 17.3; HRMS (ESI^+^): Found (M+H^+^): 416.1977 C_25_H_26_N_3_O_3_ required 416.1974.

#### (R)-1-(2-((4′-Fluoro-3-methoxy-[1,1′-biphenyl]-4-yl)oxy)propyl)-4-phenyl-1H-1,2,3-triazole (**9h**)

White amorphous solid; Yield = 89%; IR (solid): 1496, 1217, 773, 745, 669 cm^−1^; ^1^H NMR (600 MHz, chloroform-*d*) δ 8.20 (s, 1H), 7.86 (d, *J* = 7.6 Hz, 2H), 7.48 (dd, *J* = 8.3, 5.3 Hz, 2H), 7.44 (t, *J* = 7.6 Hz, 2H), 7.35 (t, *J* = 7.4 Hz, 1H), 7.11 (t, *J* = 8.5 Hz, 2H), 7.03 (d, *J* = 8.4 Hz, 2H), 6.91 (d, *J* = 8.0 Hz, 1H), 4.83–4.74 (m, 2H), 4.64 (dd, *J* = 14.5, 7.4 Hz, 1H), 3.89 (s, 3H), 1.42 (d, *J* = 6.0 Hz, 3H); ^13^C NMR (150 MHz, chloroform-*d*) δ 163.1, 161.4, 150.8, 147.5, 145.6, 136.8, 135.6, 130.6, 128.8, 128.4, 128.0, 125.6, 121.6, 119.4, 118.0, 115.6, 115.5, 111.1, 75.1, 55.8, 55.1, 17.3; ^19^F NMR (564 MHz, chloroform-*d*): δ −115.90; HRMS (ESI^+^): Found (M +H^+^): 404.1777 C_24_H_23_FN_3_O_2_ required 404.1779.

#### (R)-1-(2-((4′-Chloro-3-methoxy-[1,1′-biphenyl]-4-yl)oxy)propyl)-4-phenyl-1H-1,2,3-triazole (**9i**)

White solid; Yield = 87%; IR (solid): 1217, 772, 743, 668 cm^−1^; ^1^H NMR (600 MHz, chloroform-*d*) δ 8.15 (s, 1H), 7.81 (d, *J* = 7.6 Hz, 2H), 7.43–7.38 (m, 4H), 7.35 (d, *J* = 8.2 Hz, 2H), 7.30 (d, *J* = 14.9 Hz, 1H), 7.00 (dd, *J* = 4.2, 2.4 Hz, 2H), 6.87 (d, *J* = 8.7 Hz, 1H), 4.78–4.70 (m, 2H), 4.59 (dd, *J* = 14.4, 7.3 Hz, 1H), 3.85 (s, 3H), 1.38 (d, *J* = 6.2 Hz, 3H); ^13^C NMR (150 MHz, chloroform-*d*) δ 150.8, 147.6, 145.9, 139.1, 135.2, 133.1, 130.7, 128.8, 128.1, 128.0, 125.6, 121.5, 119.4, 117.8, 111.0, 75.1, 55.8, 55.0, 17.3; HRMS (ESI^+^): Found (M +H^+^): 420.1478 C_24_H_23_ClN_3_O_2_ required 420.1475.

#### (R)-1-(2-((2′,6′-Difluoro-3-methoxy-[1,1′-biphenyl]-4-yl)oxy)propyl)-4-phenyl-1H-1,2,3-triazole (**9j**)

Colorless amorphous solid; Yield = 91%; IR (solid): 1497, 1216, 756, 666 cm^−1^; ^1^H NMR (600 MHz, chloroform-*d*) δ 8.14 (s, 1H), 7.82–7.78 (m, 2H), 7.39 (t, *J* = 7.6 Hz, 2H), 7.29 (d, *J* = 7.5 Hz, 1H), 7.09–6.98 (m, 4H), 6.94 (dq, *J* = 8.7, 3.9 Hz, 1H), 6.88 (d, *J* = 8.2 Hz, 1H), 4.78–4.69 (m, 2H), 4.59 (dd, *J* = 14.2, 7.1 Hz, 1H), 3.83 (s, 3H), 1.38 (d, *J* = 6.2 Hz, 3H); ^13^C NMR (150 MHz, chloroform-*d*) δ 159.5, 157.8, 156.4, 154.7, 150.4, 147.6, 146.2, 130.7, 129.5, 128.8, 128.0, 125.6, 121.5, 117.2, 116.5, 115.0, 112.9, 75.0, 55.8, 54.9, 17.3; ^19^F NMR (564 MHz, chloroform-*d*): δ −119.02, 124.00; HRMS (ESI^+^): Found (M+H^+^): 422.2606 C_24_H_22_F_2_N_3_O_2_ required 422.2608.

### *In vitro* Carbonic Anhydrase-II Inhibition Assay

*In vitro* experiment of bovine erythrocyte CA-II was conducted in HEPES-Tris buffer (20 mM) to maintain the pH 7.4. The purified was dissolved in HEPES-Tris buffer (0.1 mg/ml), and the reaction mixture was comprised of 140 μL of the HEPES-Tris buffer, 20 μL of the enzyme, and 20 μL of test compound (prepared in DMSO) and was incubated for 15 min at 25°C. After completion of the preincubation, the reaction was started by adding instantly the substrate *p*-nitrophenylacetate (*P*-NPA) at a concentration of 0.7 mM and prepared in methanol. To initiate the reaction, the 96-well plate was placed in a microplate reader and continuous product formation was monitored with a one-minute time interval for 30 min at 400 nm. The assay temperature was strictly controlled and kept at 25°C. All the reaction was conducted in triplicate, and the results are presented as mean.

### Molecular Docking Protocol

The molecular docking experiment was performed on Molecular Operating Environment (MOE, 2014.14). The structures of ligands were prepared by MOE and minimized with MMFF94x force field until an RMSD gradient of 0.1 kcal mol^−1^Å^−1^ was attained, and partial charges were automatically calculated. The crystal structure of bovine carbonic anhydrase-II (PDB ID: 1V9E) was downloaded from the Protein Data Bank (https://www.rcsb.org/). Water molecules at an active site within the vicinity of 3 Å were retained and the rest of them were removed. The enzyme structure was then prepared for docking simulation using Protonate 3D option in MOE. Triangle Matcher placement method and London dG scoring function were used for docking using Zn^2+^ metal ion as a constrain for molecular docking. The best-docked pose of each compound was selected based on the binding interactions and docking score. The docking protocol was first validated by redocking the standard acetazolamide in the active site of the enzyme. The best-docked pose of the known inhibitor showed a highly negative docking score (S = −5.40 kcal/mol). The calculated RMSD between the docked and the native confirmation of sulfonamide was 2.31 Å. The docked view and binding interactions of the known active inhibitor are shown in Figure 4. The validated molecular docking protocol was then used to predict the binding pattern of the newly synthesized triazole derivatives in the active site of CA-II and to elucidate their structure-activity relationship.

## Data Availability

The original contributions presented in the study are included in the article/Supplementary Material; further inquiries can be directed to the corresponding author.
